# Early recognition of cardiac surgery-associated acute kidney injury: lack of added value of TIMP2 IGFBP7 over short-term changes in creatinine (an observational pilot study)

**DOI:** 10.1186/s12871-021-01387-6

**Published:** 2021-10-13

**Authors:** Karim Lakhal, Edith Bigot-Corbel, Emilie Sacchetto, Floris Chabrun, Thomas Senage, Lucile Figueres, Maxime Leroy, Arnaud Legrand, Bertrand Rozec

**Affiliations:** 1 Service d’Anesthésie-Réanimation, Hôpital Laënnec, Centre Hospitalier Universitaire, 44093 Nantes, France; 2 Laboratoire de Biochimie, Hôpital Laënnec, Centre Hospitalier Universitaire, 44093 Nantes, France; 3 Service de Chirurgie Cardiaque, Hôpital Laënnec, Centre Hospitalier Universitaire, 44093 Nantes, France; 4 Institut National de la Santé et de la Recherche Médicale (INSERM) n°1246, Study of Perinatal, Paediatric and Adolescent Health: Epidemiological Research and Evaluation (SPHERE) Unit, Centre National de la Recherche Scientifique (CNRS), Université de Nantes, Nantes, France; 5 Service de Néphrologie et d’Immunologie clinique, institut de transplantation urologie-néphrologie, Hôtel-Dieu, Centre Hospitalier Universitaire, 44093 Nantes, France; 6 Direction de la Recherche Clinique et de l’Innovation, Centre Hospitalier Universitaire, 44093 Nantes, France; 7 Institut du Thorax, Institut National de la Santé et de la Recherche Médicale (INSERM), Centre National de la Recherche Scientifique (CNRS), Université de Nantes, 44093 Nantes, France

**Keywords:** Acute kidney injury [MeSH], Insulin-like growth factor binding proteins/urine* [MeSH], Tissue inhibitor of Metalloproteinase-2/urine* [MeSH], Tissue inhibitor of Metalloproteinase-2 [MeSH], Creatinine/blood [MeSH], Lipocalins/metabolism* [MeSH], Cystatin C, Cardiac surgical procedures [MeSH]

## Abstract

**Background:**

For the detection of cardiac surgery-associated acute kidney injury (CS-AKI), the performance of urine tissue inhibitor of metalloproteinase 2 insulin-like growth factor-binding protein 7 (TIMP2 IGFBP7) has never been compared with that of very early changes in plasma creatinine (∆pCr). We hypothesized that, in the context of perioperative haemodilution, lack of postoperative decrease in pCr would be of honourable performance for the detection of CS-AKI. We therefore aimed at comparing these biomarkers and their kinetics (primary objective). As secondary objectives, we assessed plasma neutrophil gelatinase-associated lipocalin (pNGAL), cystatin C (pCysC) and urea (pUrea). We also determined the ability of these biomarkers to early discriminate persistent from transient CS-AKI.

**Methods:**

Patients over 75 years-old undergoing aortic valve replacement with cardiopulmonary bypass (CPB) were included in this prospective observational study. Biomarkers were measured before/after CPB and at the sixth postoperative hour (H6).

**Results:**

In 65 patients, CS-AKI occurred in 27 (42%). ∆pCr from post-CPB to H6 (∆pCr_postCPB-H6_): outperformed TIMP2 IGFBP7 at H6 and its intra- or postoperative changes: area under the receiver operating characteristic curve (AUC_ROC_) of 0.84 [95%CI:0.73–0.92] vs. ≤0.67 [95%CI:0.54–0.78], *p* ≤ 0.03. The AUC_ROC_ of pNGAL, pCysC and pUrea did not exceed 0.72 [95%CI:0.59–0.83]. Indexing biomarkers levels for blood or urine dilution did not improve their performance. Combining TIMP2 IGFBP7 and ∆pCr_postCPB-H6_ was of no evident added value over considering ∆pCr_postCPB-H6_ alone. For the early recognition of *persistent* CS-AKI, no biomarker outperformed ∆pCr_postCPB-H6_ (AUC_ROC_ = 0.69 [95%CI:0.48–0.85]).

**Conclusions:**

In this hypothesis-generating study mostly testing early detection of mild CS-AKI, there was no evident added value of the tested modern biomarkers over early minimal postoperative changes in pCr: despite the common perioperative hemodilution in the setting of cardiac surgery, if pCr failed to decline within the 6 h after CPB, the development of CS-AKI was likely. Confirmatory studies with more severe forms of CS-AKI are required.

**Supplementary Information:**

The online version contains supplementary material available at 10.1186/s12871-021-01387-6.

## Background

Cardiac surgery-associated AKI (CS-AKI) is frequent and even very mild forms -including isolated transient oliguria- are associated with negative outcomes [1–3]. Prevention, early detection and treatment of CS-AKI are therefore desirable.

The diagnosis of CS-AKI is based on plasma creatinine (pCr) increase and oliguria [4]. However, those criteria are often deemed inappropriate for an early diagnosis of CA-AKI. pCr is considered insensitive to acute changes in kidney function [5] and urine output can be misleading. Indeed, despite renal injury, normal urine output can be observed if diuretics are administered or if the concentrating mechanisms of the kidneys are impaired. Furthermore, oliguria is the appropriate response to hypovolemia without renal damage [4].

Therefore, ‘modern’ biomarkers were proposed, such as neutrophil gelatinase-associated lipocalin (NGAL), plasma cystatin C (pCysC) and, more recently, two urinary cell-cycle arrest biomarkers combined in a unique test (NEPHROCHECK®): tissue inhibitor of metalloproteinase 2 and insulin-like growth factor-binding protein 7 (TIMP2 IGFBP7) [5–7]. In a large cohort mixing patients from surgical and medical intensive care units (ICUs), TIMP2 IGFBP7 outperformed other biomarkers [8]. In the specific setting of cardiac surgery, TIMP2 IGFBP7 is considered as a valuable tool [9, 10]. Conflicting conclusions were reported for other biomarkers [6]. Surprisingly, comparisons of biomarkers performance are scarce. Only one study compared the accuracy of TIMP2 IGFBP7 with another renal biomarker (NGAL) in the setting of adults cardiac surgery [11].

Of note, pCr and its very early postoperative changes were scarcely assessed to detect CS-AKI. Owing to cardiac surgery-associated haemodilution, pCr is expected to decrease in the immediate aftermath of the procedure. Hence, lack of decrease in pCr may be an early indicator of CS-AKI. Interestingly, the two studies which assessed short-term changes in pCr after cardiac surgery reported a very encouraging diagnostic performance [12, 13]. To the best of our knowledge, no study compared TIMP2 IGFBP7 with short-term changes in pCr.

As a primary objective, we aimed to compare the performance of urine TIMP2 IGFBP7 (NEPHROCHECK®) at H6 to early postoperative variations of pCr for the detection of CS-AKI. As secondary objectives, we analyzed the performance of early changes in plasma NGAL (pNGAL), pCysC and urea (pUrea). Last, we assessed the ability of these biomarkers to discriminate persistent from transient CS-AKI.

## Methods

Ethical approval for this study was provided by the institutional review board of Nantes University Hospital (Groupe Nantais d’Ethique dans le Domaine de la Santé, GNEDS2013-01-08; Chairperson Prof F.Ballereau). Preoperative written consent was obtained from all patients during the pre-anaesthetic consultation.

### Patients

Adults over 75 years-old undergoing elective surgical aortic valve replacement under CPB were prospectively and consecutively included over 2 periods (November 2012–February 2013 and February 2016–January 2017). The time in-between was related to the need for additional funding. Eligible patients were identified on the schedule of operations, before the preoperative anesthesia consultation.

Patients were not included in the event of preoperative renal replacement therapy (RRT), additional surgical procedure (along with the aortic valve replacement) or refusal to participate.

### Perioperative care

Total intravenous anaesthesia (sufentanil and propofol), atracurium, cefuroxime and unfractioned heparin were administered. Fluids used for volume expansion were crystalloids (0.9% saline or Ringer lactate) and gelatin solutions. No patient received hydroxyethylstarch. CPB prime solution was Ringer lactate. All patients were transported to the ICU immediately after the completion of the surgical procedure.

### Measurements

Urine (via the dedicated port of the Foley catheter, in BD Vacutainer® Plus urinalysis tube, BD Diagnostics, Le Pont de Claix, France) and blood (in lithium heparin tube, BD Vacutainer®) were sampled before CPB initiation (pre-CPB, just after the insertion of the Foley catheter), immediately after its end (post-CPB), 6 h later (H6) and the day after the surgery (Day1).

As current practice, pUrea and pCr measurements were provided to the attending physician, contrary to other biomarkers which were measured later on, on centrifuged samples [2200 g during 10 min] frozen and stored at − 80 °C.

TIMP2 IGFBP7 was measured using an immunoassay (NEPHROCHECK®_,_ Astute Medical, San Diego, CA, USA). Other measurements were made on Cobas 6000 Roche analyser: pCr and pUrea using an enzymatic assay (Creatininase and Urease, respectively, Roche), pNGAL (ST001CA Eurobio Bioporto, Roche) and pCysC (Tina quant Cystatine Gen2, Roche) using an immunoturbidimetric assay, albuminemia using the bromocresol green method (Roche).

### Statistical analysis

#### Main outcome: CS-AKI

CS-AKI was defined according to the stage ≥1 of the Kidney Disease Improving Global Outcome (KDIGO) guidelines: within 2 days, increase in pCr of ≥26.5 μmol/L (≥0.3 mg/dL) and/or ≥ 1.5 x baseline pCr and/or urine volume < 0.5 mL/kg/h for 6 h [4]. Stage ≥2 CS-AKI was defined as a doubling of baseline pCr and/or urine output < 0.5 mL/kg/h for ≥12 h [4]. Pre-CPB pCr was baseline pCr. All pCr available measurements within the 48 postoperative hours were analyzed for the CS-AKI outcome and the worst change in pCr was considered. Urine output was assessed hourly in the ICU and every 2 to 6 h in the ward (from Day1, if permitted by the patient’s condition). Among relevant clinical and biological data (Table 1), the need for RRT during the hospital stay was collected.

**Table 1:** Patients’ characteristics

	***CS-AKI*** ***n=27 (42 %) patients***	***No CS-AKI*** ***n=38 (58 %) patients***	***p***
Age (years)	78 [75-86]	79 [75-86]	0.14
Female gender (n [%])	12 (44 %)	21 (55 %)	0.39
Body mass index (kg/m^2^)	27 [25-30]	26 [23-27]	0.04
Weight (kg)	75 [67-83]	69 [59-79]	0.06
EuroSCORE II (%)	3.4 [2.0-5.5]	1.8 [1.4-3.5]	0.016
SAPS II	27 [24-32]	24 [22-27]	0.006
Comorbidities			
Hypertension (n [%])	17 (63 %)	28 (76 %)	0.27
Diabetes mellitus (n [%])	5 (19 %)	9 (23 %)	0.58
COPD (n [%])	2 (7 %)	1 (3 %)	0.38
History of heart surgery (n [%])	1 (4 %)	1 (3 %)	0.82
Preoperative LV ejection fraction <35 % (n [%])	1 (4 %)	0 (0 %)	0.24
History of peripheral vascular surgery (n [%])	3 (11 %)	2 (5 %)	0.40
Preoperative serum creatinine (μmol/L)	82 [74-91]	79 [66-92]	0.34
Preoperative eGFR (mL/minute/1.73 m^2^)	75 [54-83]	70 [64-81]	0.85
eGFR before CPB <60 mL/min/1.73 m^2^	7 (26 %)	5 (13 %)	0.38
Cross-clamp time (minutes)	45 [38-58]	56 [43-68]	0.014
CPB time (minutes)	61 [53-76]	68 [56-89]	0.09
CPB priming (mL)	1000 [1000-1100]	1000 [1000-1025]	0.79
Volume expansion (mL)			
Intraoperative	850 [500-1100]	600 [500-1000]	0.33
Post-operative (24h)	750 [500-1250]	500 [250-875]	0.046
Intraoperative transfusion			
Red blood cell autotransfusion (mL)	400 [210-500]	400 [300-500]	0.85
Packed red blood cell (n patients)	6 (22 %)	13 (35 %)	0.35
Packed red blood cell >2 packs (n patients)	1 (4 %)	0 (0 %)	0.24
Fresh frozen plasma (n patients)	1 (4 %)	0 (0 %)	0.24
Platelet transfusion (n patients)	1 (3 %)	1 (4 %)	0.82
Postoperative (48h) transfusion			
Packed red blood cell (n patients)	6 (22 %)	4 (11 %)	0.22
Packed red blood cell > 2 packs (n patients)	1 (4%)	0 (0 %)	0.24
Fresh frozen plasma (n patients)	1 (4 %)	1 (3 %)	0.82
Platelet transfusion (n patients)	1 (4 %)	0 (0 %)	0.24
Post-operative (24 h) urine output (mL/kg/h)	0.7 [0.5-0.9]	1.0 [0.7-1.3]	0.003
Diuretics within 24 postoperative hours (n [%])	19 (70 %)	14 (38 %)	0.010
Vancomycin for antibioprophylaxis (n [%])	0 (0 %)	0 (0 %)	-
Aminoglycosides within 48 postop. hours (n [%])	0 (0 %)	1 (3 %)	0.39
Re-operation within 48 hours	2 (7 %)	0 (0 %)	0.10
ICU length of stay (days)	3 [2-5]	2 [1-3]	0.037
Hospital length of stay after the surgery (days)	10 [8-14]	9 [8-13]	0.37
Hospital mortality (n [%])	0 (0 %)	1 (3 %)	0.39

*eGFR* estimated glomerular filtration rate (modification of diet in renal disease equation), *COPD* Chronic obstructive pulmonary disease, *ICU* intensive care unit, *SAPS II* simplified acute physiology score 2, *LV* left ventricle, *CPB* cardiopulmonary bypass

Results are expressed as n (%) or median [interquartile range]

#### Comparison of biomarkers performance

The area under the receiver operating characteristic curve (AUC_ROC_) was determined for either isolated measurements or concentration changes between two time points. AUC_ROC_s were compared [14]. Sensitivity, specificity, positive and negative predictive values were determined. For TIMP2 IGFBP7, the proposed threshold of 0.3 (ng/mL)^2^/1000 was tested [7].

#### Analyses for secondary objectives

We explored whether correcting the levels of plasma biomarkers for hemodilution (a frequent condition during and after cardiac surgery with CPB) improved their diagnostic performance. Change in albuminemia was used for this purpose. Example:

$$ corrected\ pNGAL\  at\ H6= pNGAL\  at\ H6+\left[ pNGAL\  at\ H6\ x\ \left(\% decrease\ in\ albuminemia\ between\  pre- CPB\  and\ H6\right)\right] $$

Similarly, the degree of concentration of urines may be altered (by excessive volume expansion, diuretics use for instance). To overcome this confounding factor, the performance of the ratio of urine TIMP2 IGFBP7 level to urine creatinine was assessed.

The diagnostic performances of TIMP2 IGFBP7, pNGAL, pCysC, pUrea at day 1 were also tested.

Combinations of TIMP2 IGFBP7 and changes in pCr were assessed. Cutoffs were determined by the Youden index.

Last, among patients developing CS-AKI, we assessed the ability of the biomarkers measured at H6 or earlier to discriminate between persistent and transient CS-AKI. Only for this very specific analysis, the KDIGO stage of CS-AKI determined from H48 to H60 were compared to that determined before H48. Transient CS-AKI was defined as a decrease in the KDIGO stage over time. Otherwise, CS-AKI was considered persistent [15].

Between-groups comparisons relied on chi-squared and Mann-Whitney tests. A *p* < 0.05 was considered significant. Analysis was performed on anonymous data with MedCalc*™*15.8 (MedCalc Software bv, Ostend, Belgium). No data imputation was performed.

This manuscript is in accordance with the STARD statements for the reporting of studies of diagnostic accuracy (Supplemental Table 1) [16].

#### Study size

The funding capacity for this pilot study allowed measurements of TIMP2 IGFBP7 in 65 patients. Lack of timely intra-or postoperative blood or urine sampling was expected in some patients since inclusion was made during the preoperative anaesthesia consultation and the perioperative availability of the investigators was not guaranteed. We therefore included patients until reaching a total of 65 patients with measurements of TIMP2 IGFBP7 at H6 and plasma creatinine. This study size permits to detect a difference of 0.20 (α = 0.05 and β = 0.20) between the AUC_ROC_ of postoperative change in pCr (a priori unknown) and that of TIMP2 IGFBP7 at H6 (assumed to be ≈0.85 [9, 17]), assuming a correlation between these two biomarkers of 0.50 and an incidence of CS-AKI of 40% [1, 2].

## Results

**Fig. 1:**
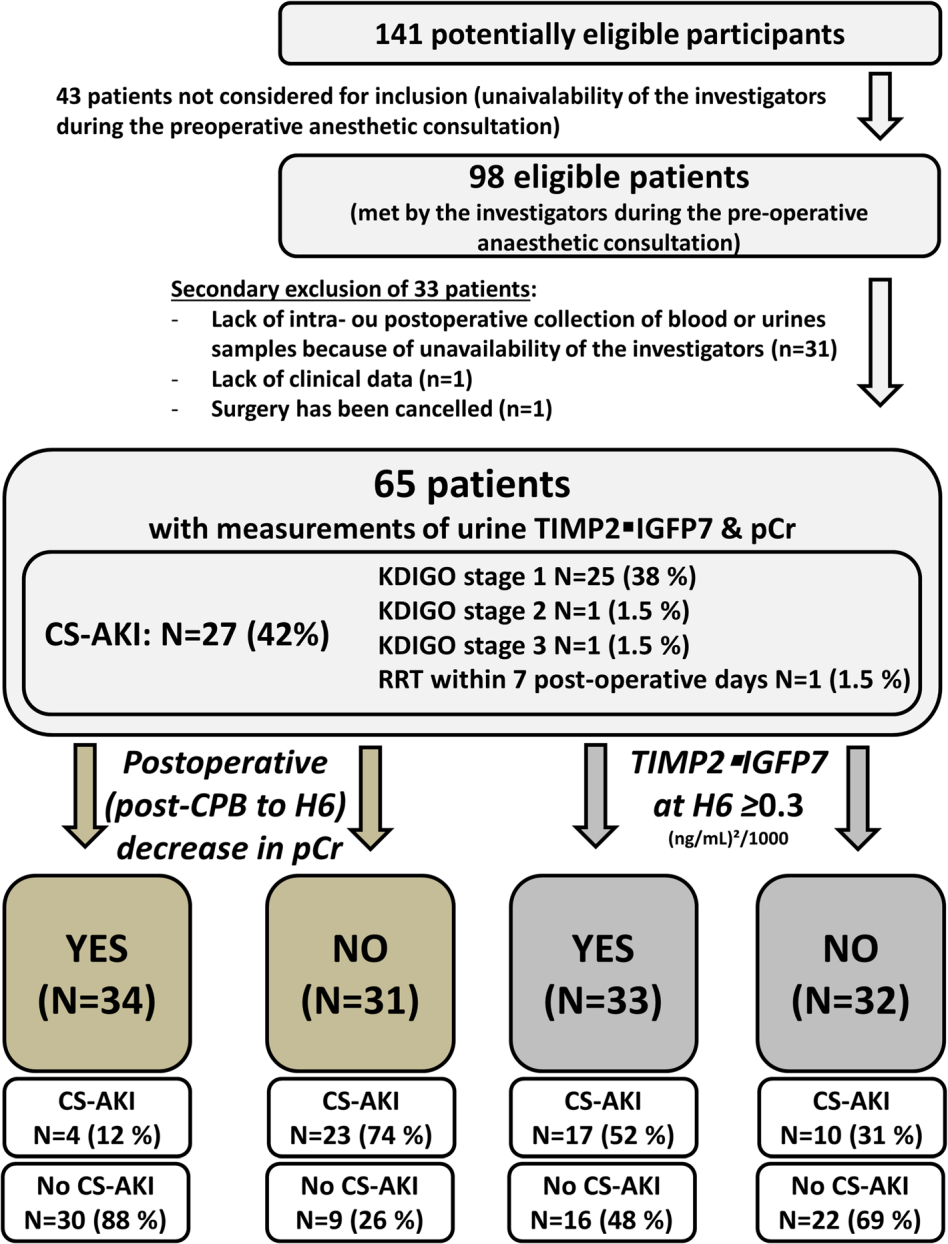
Study diagram. Legend: This diagram follows STARD reporting guideline for diagnostic accuracy studies. CS-AKI: cardiac surgery-associated acute kidney injury; CPB: cardiopulmonary bypass; pCr: plasma creatinine; TIMP2 IGFBP7: tissue inhibitor of metalloproteinase 2 – insulin-like growth factor-binding protein 7; RRT: renal replacement therapy. CS-AKI was classified according to Kidney Disease Improving Global Outcome (KDIGO) guidelines including both pCr change and the urine output criterion

Among 98 included patients, 65 had measurements of both TIMP2 IGFBP7 and pCr (Table 1). Reasons for exclusion (mostly related to investigators unavailability) are depicted in Fig. 1. Baseline characteristics of excluded patients did not differ from those of included patients (Supplemental Table 2).

Twenty seven patients (42%) developed CS-AKI, mostly stage 1 CS-AKI (25 patients [93%], Fig. 1). One patient (1.7%) needed RRT within the week after CPB. CA-AKI patients had a longer length of stay in the ICU (3 [IQR 2–5] vs. 2 [1, 2, 4] days; *p* = 0.037).

### Primary objective: comparison of TIMP2 IGFBP7 and pCr for the early detection of CS-AKI (*n* = 65 patients)

**Fig. 2:**
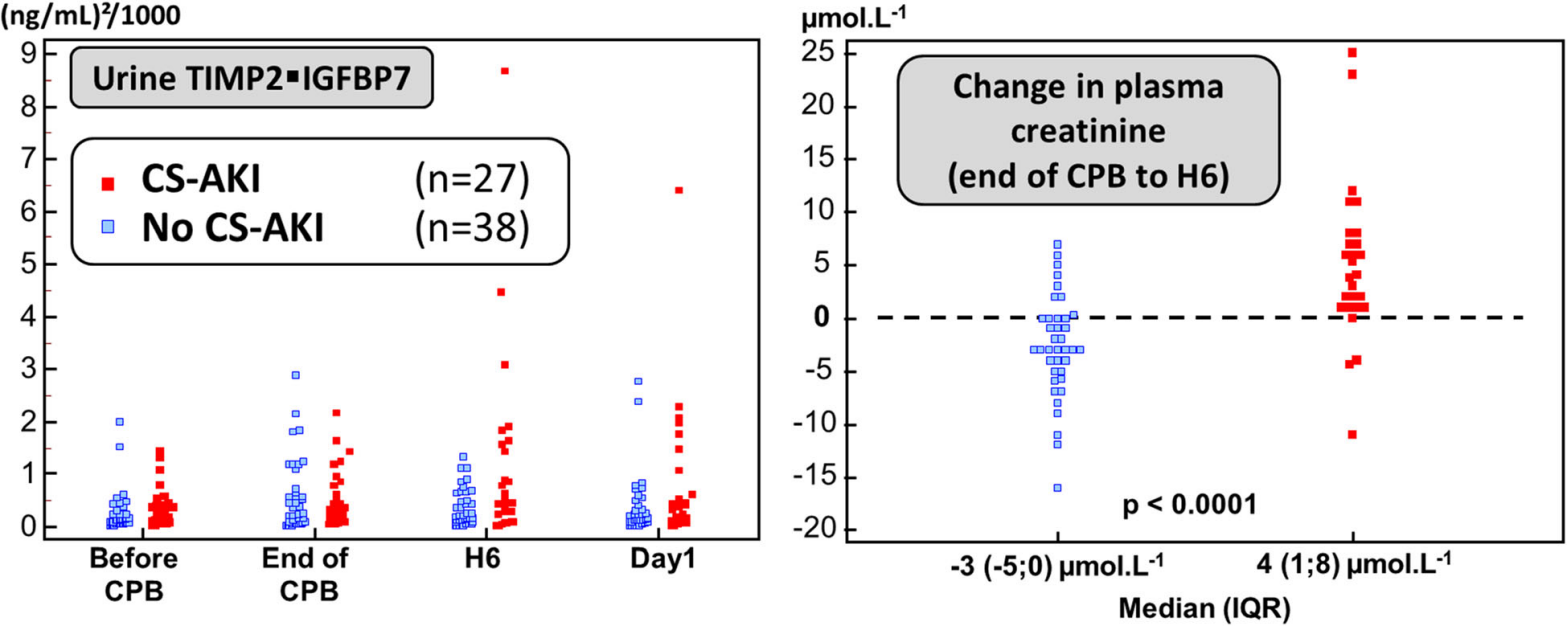
Concentration of TIMP2 IGFBP7 and change in pCr. Legend: CS-AKI: Cardiac surgery-associated acute kidney injury (CS-AKI); TIMP2 IGFBP7: tissue inhibitor of metalloproteinase 2 – insulin-like growth factor-binding protein 7; pCr: plasma creatinine. CPB: cardiopulmonary bypass

There was a marked overlap between TIMP2 IGFBP7 measurements at H6 in patients who developed CS-AKI and those who did not (Fig. 2) and the associated AUC_ROC_ to detect CS-AKI was only fair (AUC_ROC_ = 0.64 [95%CI 0.51;0.76]). The commonly proposed cutoff of 0.3 (ng/mL)^2^/1000 for TIMP2 IGFBP7 was associated, at H6, with poor sensitivity and specificity (63% [95%CI 42–81%] and 58% [95%CI 41–74%], respectively).

**Fig. 3:**
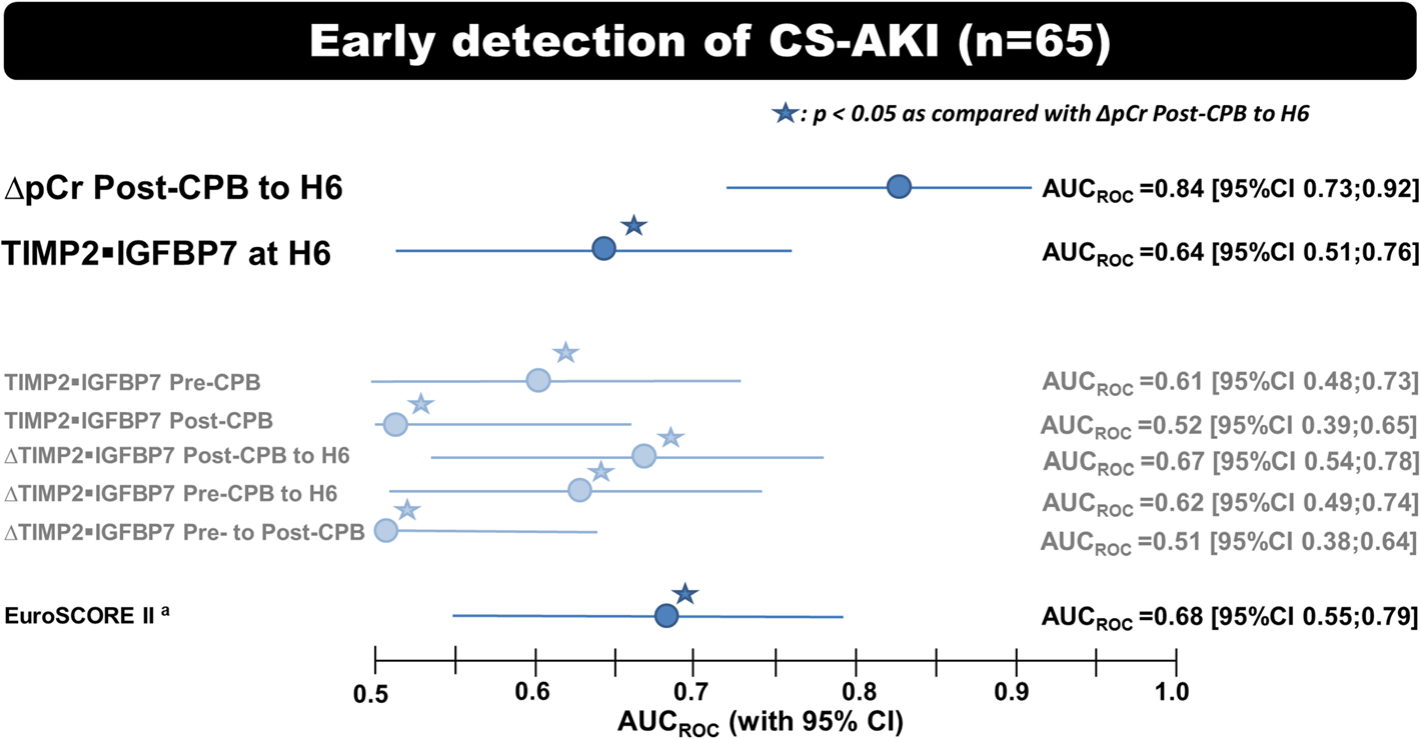
Early recognition of cardiac surgery-associated AKI (*n* = 65 patients). Legend: The accuracy for the prediction or the detection of stage ≥1 cardiac surgery-associated acute kidney injury (CS-AKI) was assessed via the area under receiver operating characteristic curve (AUC_ROC_ [95% confidence interval]). Each biomarker was tested for 1) an isolated sample taken before (Pre-CPB), immediately after (Post-CPB) cardiopulmonary by-pass and 6 h (H6) later and 2) for change in concentration between 2 time points. ∆: change in biomarker concentration; TIMP2 IGFBP7: tissue inhibitor of metalloproteinase 2 – insulin-like growth factor-binding protein 7; pCr: plasma creatinine

Whether we considered changes between two time points or isolated measurements, the AUC_ROC_ of TIMP2 IGFBP7 was below 0.68, significantly lower (*p* ≤ 0.03) than the AUC_ROC_ of absolute changes in pCr from post-CPB to H6 (∆pCr_postCPB-H6_): AUC_ROC_ = 0.84 (95%CI 0.73–0.92) (Fig. 3). Lack of postoperative decrease in pCr (∆pCr_postCPB-H6_ > 0 μmol. L^− 1^) was associated with 85% (95%CI 66–96%) and 79% (95%CI 63–90%) sensitivity and specificity, respectively.

Considering percentage rather than absolute (in μmol. L^− 1^) change in pCr did not improve the performance of ∆pCr_postCPB-H6_: AUC_ROC_ = 0.82 (95%CI 0.71–0.91) vs. 0.84 (95%CI 0.73–0.92), respectively (*p* = 0.8). Likewise, considering changes in pCr from pre-CPB (rather than post-CPB) to H6 levels was not superior to ∆pCr_postCPB-H6_ (AUC_ROC_ = 0.68 [95%CI 0.56–0.79] vs.0.84 [0.73–0.92]; *p* = 0.07).

### Secondary objectives

#### Comparison of all biomarkers for the early detection of CS-AKI (*n* = 59 patients)

In 59 out of 65 patients, all biomarkers, including pNGAL, pCysC, and pUrea, were measured at all time points. Twenty-six of these patients (44%) developed CS-AKI.

For pNGAL, pCysC and pUrea, as for TIMP2 IGFBP7, there was a marked overlap of the concentrations measured in patients who developed CS-AKI and those who did not (Supplemental Figure 1).

**Fig. 4:**
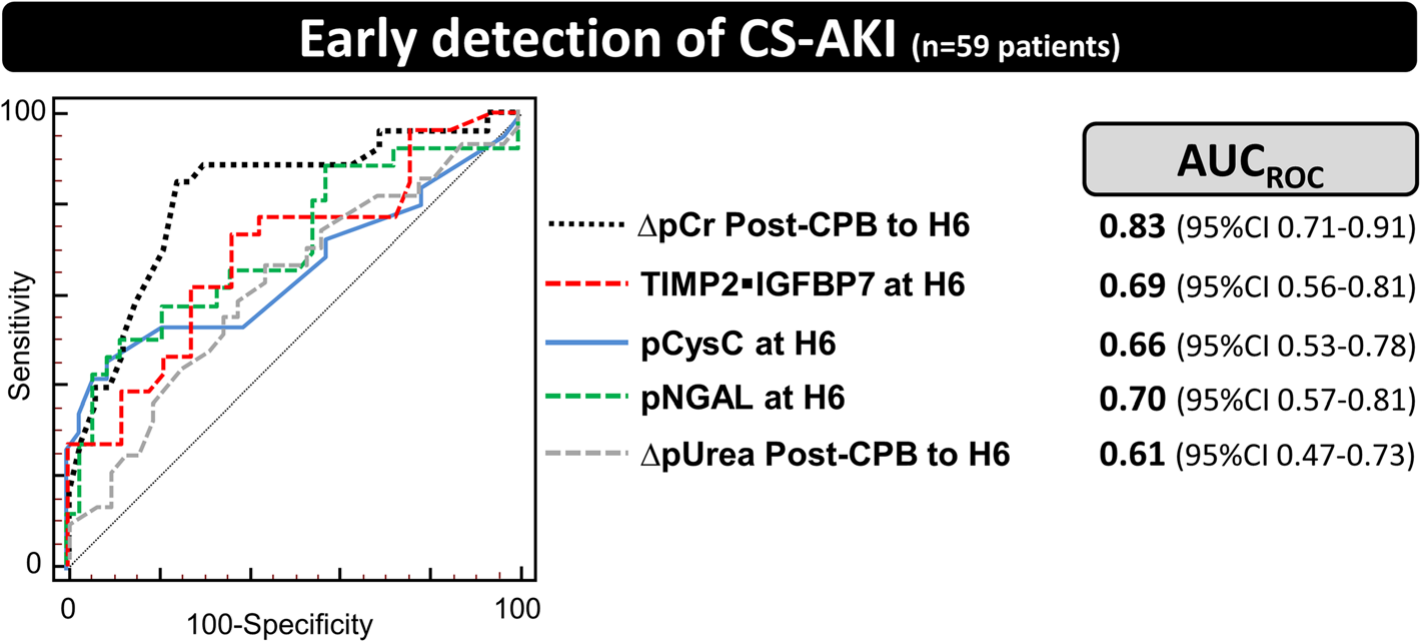
Early recognition of cardiac surgery-associated AKI in patients in whom all biomarkers were measured (*n* = 59). Legend: The accuracy for the prediction or the detection of stage ≥1 cardiac surgery-associated acute kidney injury (CS-AKI) was assessed via the area under receiver operating characteristic curve (AUC_ROC_ [95% confidence interval]). Twenty-six patients (44%) developed CS-AKI (mostly stage 1 CS-AKI, only 2 patients developing stage 2–3). CPB: cardiopulmonary bypass; ∆: change in biomarker concentration; TIMP2 IGFBP7: tissue inhibitor of metalloproteinase 2 – insulin-like growth factor-binding protein 7; pCr: plasma creatinine; NGAL: neutrophil gelatinase-associated lipocalin

Whether we considered changes between two time points or isolated measurements, the AUC_ROC_ of these biomarkers was not higher than that of ∆pCr_postCPB-H6_ (Fig. 4, Supplemental Figure 2 and Supplemental Tables 3a to e).

Biomarkers performance was not enhanced by:

- *correcting plasma biomarkers measurements for hemodilution* by indexing them to change in albuminemia (Supplemental Tables 3a-e);
- *indexing urine TIMP2 IGFBP7 to urine creatinine* (at H6: AUC_ROC_ = 0.63 [95%CI 0.50–0.75]);
- *considering late (day 1) measurements* of TIMP2 IGFBP7, pNGAL, pCysC or pUrea (Supplemental Tables 3b-e): AUC_ROC_ = 0.59 [95%CI 0.46–0.71] for TIMP2 IGFBP7 at day 1 (*n* = 61);

Likewise, omitting the urine output criterion of the CS-AKI definition did not change the conclusions (Supplemental Table 4).

#### Combination of biomarkers: refining pCr-based early detection of CS-AKI with the use of a second biomarker

Thrity-one patients experienced lack of postoperative decrease in pCr (positive ∆pCr_postCPB-H6_ test) and therefore had high likelihood for developping CS-AKI. In these patients, we assessed whether the adjunction of TIMP2 IGFBP7 could be helpful to discriminate between false and true positive cases for ∆pCr_postCPB-H6_. Median TIMP2 IGFBP7 at H6 was of no added value since it was not significantly different in patients who developed CS-AKI (*n* = 23 true positives for ∆pCr_postCPB-H6_) and those who did not (*n* = 8 false positives): 0.42 (IQR 0.20;0.81) versus 0.45 (IQR 0.09;1.56) (ng/mL)^2^/1000; *p* = 0.70.

**Fig. 5:**
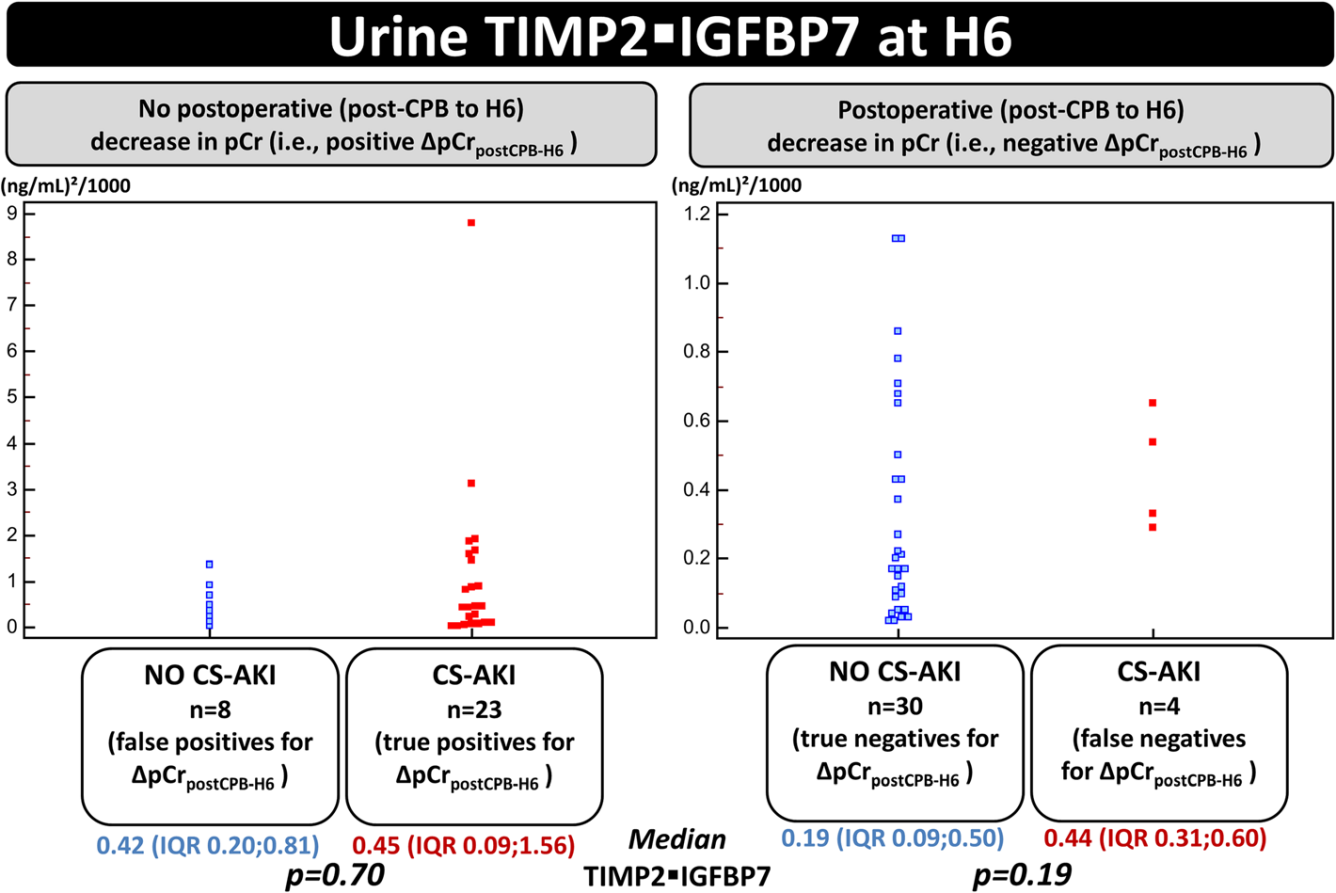
Combination of pCr and TIMP2 IGFBP7 for the early detection of CS-AKI. Legend: CS-AKI: Cardiac surgery-associated acute kidney injury; CPB: cardiopulmonary bypass; ∆pCr_postCPB-H6_: change in plasma creatinine from CPB to H6; TIMP2 IGFBP7: tissue inhibitor of metalloproteinase 2 – insulin-like growth factor-binding protein 7. CS-AKI was classified according to Kidney Disease Improving Global Outcome (KDIGO) guidelines. For each panel, the marked overlap between values TIMP2 IGFBP7 in patients who developed CS-AKI and those who did not suggests that TIMP2 IGFBP7 at H6 was of no evident added value to ∆pCr_postCPB-H6_ for a better stratification of the risk of CS-AKI as compared with the use of ∆pCr_postCPB-H6_ alone

Likewise, in patients in whom ∆pCr_postCPB-H6_ indicated low likelihood of developing CS-AKI (∆pCr_postCPB-H6_ ≤ 0 μmol. L^− 1^), TIMP2 IGFBP7 was of little help to discriminate between true (*n* = 30) and false *negative* cases (*n* = 4) for ∆pCr_postCPB-H6_: median TIMP2 IGFBP7 at H6 of 0.19 (IQR 0.09;0.50) versus 0.44 (IQR 0.31;0.60) (ng/mL)^2^/1000; *p* = 0.19 (Fig. 5). For pUrea or its changes, these conclusions were similar (data not shown).

A high level of pNGAL (or pCysC to a lesser extent) may be helpful to distinguish between false and true positive cases and may therefore improve the biomarkers-based risk stratification of developing CS-AKI. However, wide confidence intervals prevent drawing firm conclusions (Supplemental Figure 3a to c).

*The ability to detect stage 2–3 CS-AKI* was not analyzed since only 2 patients developed it.

#### Early discrimination of persistent from transient CS-AKI

TIMP2 IGFBP7, pNGAL, or pCysC did not outperform ∆pCr_postCPB-H6_ for this purpose (AUC_ROC_ = 0.69 [95%CI:0.48 to 0.85] (Supplemental Figure 4).

## Discussion

In patients over 75 years-old undergoing surgical aortic valve replacement with CPB and experiencing CS-AKI with an incidence which is in line with recent studies using the same modern definition [1–3, 18], ∆pCr_postCPB-H6_ outperformed TIMP2 IGFBP7 at H6 for the early recognition of CS-AKI. TIMP2 IGFBP7 measured at other timepoints were not of higher performance, as well as changes in TIMP2 IGFBP7. Similar findings were observed for pNGAL, CysC and pUrea. To discriminate persistent from transient CS-AKI, no novel biomarker outperformed pCr.

As in the present study, a limited performance has been previously reported for pNGAL and CysC after cardiac surgery [12]. In various settings, urine TIMP2 IGFBP7 is considered to be the most accurate biomarker [19]. In cardiac surgery, a recent meta-analysis of 10 studies found that the pooled AUC_ROC_ of TIMP2 IGFBP7 for the detection of CS-AKI was remarkable (AUC_ROC_ = 0.83 [standard error 0.02]) [9]. So why did not we find such good diagnostic accuracy for TIMP2 IGFBP7? Firstly, not all the available studies reported good performance for TIMP2 IGFBP7: some recent studies, not included in Su et al. meta-analysis, reported, as we did, a poor-to-moderate performance for TIMP2 IGFBP7 (AUC_ROC_ = 0.57–0.69) [20–22]. Secondly, Day1 measurement of TIMP2 IGFBP7 has been included in this meta-analysis [9]. For instance, in one study, an AUC_ROC_ of TIMP2 IGFBP7 at Day1 (0.71) has been retained whereas AUC_ROC_s at the end of the surgery (AUC_ROC_ = 0.63) or at the 4th hour (AUC_ROC_ = 0.51) have not [23]. However, beyond the fact that, at Day1, the superiority of TIMP2 IGFBP7 over pCr is highly uncertain, we believe that Day1 is a time point which is too late to provide nephroprotective interventions and is therefore not an *early diagnosis* of CS-AKI. Thirdly, several studies defined CS-AKI only by pCr increase, i.e., omitted the urine output criterion [24–29]. Yet, even possibly misleading, urine output is full part of the recommended definition of AKI [4]. Last, in 3 studies included in Su et al.’s meta-analysis, TIMP2 IGFBP7 has been analyzed only for the detection of stage 2–3 CS-AKI [9] and in most other studies, stage 2–3 CS-AKI featured prominently among cases of CS-AKI. This may have contributed to the good pooled performance of TIMP2 IGFBP7 for the detection of all stages CS-AKI. Indeed, the more severe the disease, the more sensitive and specific its biomarker. Hence, the diagnostic performance of TIMP2 IGFBP7 is probably better for the identification of moderate-to-severe CS-AKI (stage 2–3) than for mild CS-AKI (stage 1). It is noteworthy that TIMP2 IGFBP7 has been approved by the US Food and Drug Administration as an aid in the risk assessment for moderate or severe (stage 2–3) acute kidney injury within the next 12 h. Our study, in which most (92%) CS-AKI cases were mild (stage 1), contrary to several previous studies [9], discourages to extend the indication for TIMP2 IGFBP7 to the detection of *mild* CS-AKI developing within the 48 postoperative hours.

Our results challenges the long-held belief that pCr is too slow in its response to renal injury to provide a valuable prediction of adverse outcomes. However, our findings are in line with those of the scarce studies assessing the performance of early measurements of pCr. Indeed, minimal very short-term changes in pCr were associated with 30-day mortality after cardiac surgery [30] or with a composite outcome of hospital mortality or need for RRT [13]. Closer to the scope of the present study, i.e., early detection of CS-AKI, Ho et al. reported the utility of an early postoperative increase in pCr by ≥10% from baseline (odds ratio = 6.4 [95%CI:2.37–17.2]) [31]. McIlroy et al. found that if early postoperative pCr measurements failed to decline, the occurrence of CS-AKI was likely (odds ratio = 2.6 [95%CI 1.7–4.1]) [13]. These findings are reinforced by the present study, which, besides, provides the AUC_ROC_ for the CS-AKI outcome. We found the same threshold as that proposed by McIllroy et al. for change in pCr (0 μmol. L^− 1^) [13], in spite of the fact that we considered change in pCr from post-CPB to H6 (vs. from preoperative pCr to ≈ 3 h of ICU admission). Hence, minimal very short-term changes in pCr may reflect renal injury. Owing to perioperative volume expansion (especially during cardiac surgery with CPB), a decrease in pCr level is expected in the immediate aftermath of the procedure. Several hypotheses may be advanced if pCr fails to decline: reduced renal creatinine clearance, increased perioperative creatinine production, reduction in total body water (related to diuretic administration for instance) [13, 31] or misleading measurements of pCr owing to analytical variability. However, the analytical precision of the enzymatic assay we used for pCr was good in our laboratory during the study period and beyond: maximum coefficient of variation of only 2.9% (even lower for elevated levels of pCr).

### Strengths of this study

The present study is prospective and, again, used the full current definition of AKI [4]. It compared multiple modern biomarkers within the same population. Of note, only one study in adult patients undergoing cardiac surgery compared TIMP2 IGFBP7 to another novel biomarker (urine NGAL) [11]. Two studies did so in children, testing urine NGAL and Kidney Injury Molecule-1 [11, 27]. In addition to TIMP2 IGFBP7, pNGAL and pCysC, and contrary to the vast majority of previous studies, we also reported the ability of early measurements of pCr and pUrea for the detection of CS-AKI. Our comprehensive analysis encompassed isolated measurements of biomarkers and also their kinetics. We explored the impact of the indexation of biomarkers levels to changes in albuminemia (as a gauge of haemodilution) or to urine creatinine (to overcome the heterogeneity in urine concentration). Last, contrary to previous studies in the cardiac surgery setting, we distinguished the persistent and the transient nature of CS-AKI according to recent recommendations [15].

### Study limitations

First, our findings observed in 65 patients, should be validated in a larger cohort. A larger study size is required for a more robust analysis of biomarkers combinations and a specific analysis of stage 2–3 CS-AKI detection. Additional studies will also be needed to assess whether a biomarker-guided implementation of nephroprotective measures at the 6th hour after the end of CPB (plus the turnaround time for pCr) positively impacts patients outcome. Of note, this has been found with TIMP2 IGBP7 measurements at the 4th hour [36].

Second, owing to the exploratory nature of most analyses, we preferred preventing potentially useful findings not being overlooked [32]. We therefore did not make adjustments for multiple tests but we cannot exclude that some significant differences we found were actually due to chance.

Third, only elderly patients (≥ 75 years-old) undergoing aortic valve replacement with CPB were included. Caution should therefore be exercised before any extrapolation to other populations. We chose to focus on this specific population because, in these often fragile patients, a less invasive alternative therapy for aortic valve stenosis is increasingly proposed when cardiac surgery with CPB is of questionable benefit/risk ratio: transcatheter aortic valve implantation [34]. To optimize the safety of the open surgery option, reliable renal biomarkers are required to prompt nephroprotective measures [4, 19].

Fourth, we cannot exclude that some false negative cases for TIMP2 IGFBP7 (or other biomarkers) were actually true negatives: some stage 1 CS-AKI cases could have been “functional”, i.e.*,* with no kidney cell damage, explaining the lack of increase in novel biomarkers levels in patients with mild increase in pCr and/or oliguria [9]. However, “*even mild reversible AKI has important clinical consequences*” as stated in KDIGO guidelines and supported by several studies in the setting of cardiac surgery [1, 2, 12, 13, 30, 33, 35], and should therefore be detected as early as possible [4, 33].

Fifth, we did not strictly use all the criteria of the KDIGO definition. Indeed, we focused on the renal impact of the index cardiac surgery procedure (and very early potential complications) rather than later complications. As other authors [1, 3, 36], we therefore arbitrarily reduced the observation period of the CS-AKI definition. We used a 48 h-time window for all criteria although one of the three criteria of the KDIGO definition (relative increase in pCr) has been proposed with a 7-day time window [4]. Enlarging the time window, i.e., using a more sensitive definition, could have yielded higher incidence of CS-AKI and, possibly, different performance of the tested biomarkers.

Last, pCr was both biomarker under-test and full part of the definition (within 48 postoperative hours, along with oliguria) of the outcome measure (CS-AKI). Although only early (H6 or earlier) measurements of pCr were analyzed for the early detection of CS-AKI, pCr may therefore be seen as both judge and judged. Again, we used the recommended definition of AKI [4]. Confirming our findings by using a definition of CS-AKI fully unrelated to pCr would be desirable, but such robust standard does not exist to date.

## Conclusions

In summary, in a population with low incidence of stage 2–3 CS-AKI, there was no evident added value of TIMP2 IGFBP7 at H6 over ∆pCr_postCPB-H6_ for the early recognition of mild (stage 1) CS-AKI and of it persistent nature. Despite the common perioperative hemodilution in the setting of cardiac surgery, if pCr failed to decline within the 6 h after CPB, the development of CS-AKI was likely. These findings should be confirmed for both internal and external validity and cannot be extrapolated to patients with more severe forms of CS-AKI.

## Supplementary Information

**Additional file 1: Supplemental Figure 1.** Concentration of biomarkers in the same population. **Supplemental Figure 2.** Early detection of CS-AKI in the same population. **Supplemental Figure 3.** Refining pCr-based early detection of CS-AKI with the use of a second biomarker. **Supplemental Figure 4.** Distinction between persistent and transient cardiac surgery-associated AKI. **Supplemental Table 1.** STARD checklist. **Supplemental Table 2.** Comparison of baseline characteristics of included and excluded patients. **Supplemental Table 3a to 3e.** Performance of biomarkers for the detection of CS-AKI. **Supplemental Table 4.** Performance for the prediction or detection of CS-AKI according to the definition (omitting the urine output criterion or not).

## Data Availability

The datasets used and/or analysed during the current study are available from the corresponding author on reasonable request.

## Abbreviations

95%CI: 95% confidence interval
AUC_ROC_: Area under the receiver operating characteristic curve
CPB: Cardiopulmonary bypass
CS-AKI: Cardiac surgery-associated AKI
ICU: Intensive care unit
KDIGO: Kidney Disease Improving Global Outcome
NGAL: Neutrophil gelatinase-associated lipocalin
pCr: plasma creatinine
pCysC: plasma cystatin C
pUrea: plasma urea
pNGAL: plasma NGAL
RRT: Renal replacement therapy
TIMP2 IGFBP7: Tissue inhibitor of metalloproteinase 2 & insulin-like growth factor-binding protein 7
