# Supplementary material for: Early recognition of cardiac surgery-associated acute kidney injury: lack of added value of TIMP2 IGFBP7 over short-term changes in creatinine (an observational pilot study)
Source: BMC Anesthesiol. 2021 Oct 13;21:244. doi: 10.1186/s12871-021-01387-6 (PMC8513334; doi:10.1186/s12871-021-01387-6)
Supplement: Supplementary file 1 — Additional file 1: Supplemental Figure 1. Concentration of biomarkers in the same population. Supplemental Figure 2. Early detection of CS-AKI in the same population. Supplemental Figure 3. Refining pCr-based early detection of CS-AKI with the use of a second biomarker. Supplemental Figure 4. Distinction between persistent and transient cardiac surgery-associated AKI. Supplemental Table 1. STARD checklist. Supplemental Table 2. Comparison of baseline characteristics of included and excluded patients. Supplemental Table 3a to 3e. Performance of biomarkers for the detection of CS-AKI. Supplemental Table 4. Performance for the prediction or detection of CS-AKI according to the definition (omitting the urine output criterion or not). [file 12871_2021_1387_MOESM1_ESM.doc]

***Early recognition of cardiac surgery-associated acute kidney injury: lack of added value of TIMP2▪IGFBP7 over short-term changes in creatinine (an observational pilot study).***

***SUPPLEMENTAL MATERIAL***

Supplemental Figure 1: Concentration of biomarkers in the same population.

Supplemental Figure 2: Early detection of CS-AKI in the same population.

Supplemental Figure 3: Refining pCr-based early detection of CS-AKI with the use of a second biomarker.

Supplemental Figure 4: Distinction between persistent and transient cardiac surgery-associated AKI.

Supplemental Table 1: STARD checklist.

Supplemental Table 2: Comparison of baseline characteristics of included and excluded patients.

Supplemental Table 3a to 3e: Performance of biomarkers for the detection of CS-AKI.

Supplemental Table 4: Performance for the prediction or detection of CS-AKI according to the definition (omitting the urine output criterion or not).

**Supplemental Figure 1: Concentration of biomarkers.**


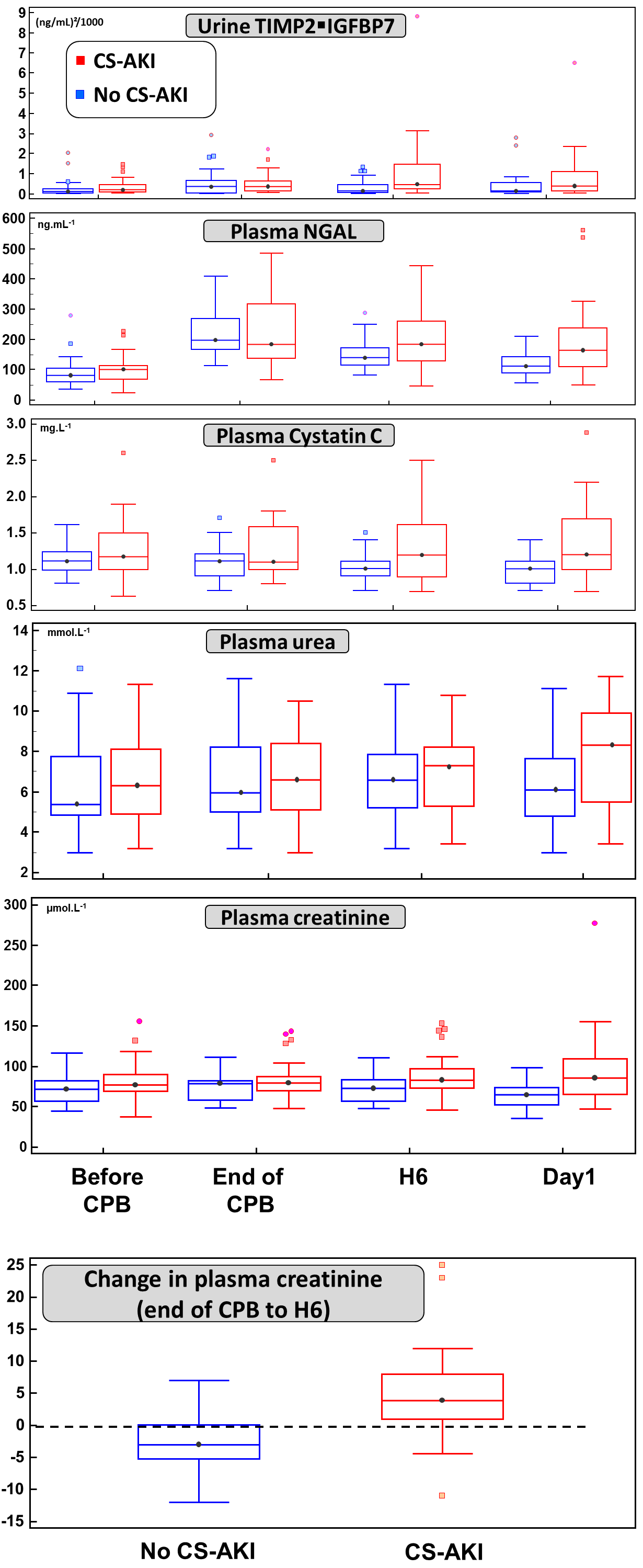


Legend: CS-AKI: Cardiac surgery-associated acute kidney injury (CS-AKI); TIMP2▪IGFBP7: tissue inhibitor of metalloproteinase 2 ̶ insulin-like growth factor-binding protein 7; NGAL: neutrophil gelatinase-associated lipocalin;

CS-AKI was classified according to Kidney Disease Improving Global Outcome (KDIGO) guidelines. Patients with stage 2-3 CS-AKI were not included in this analysis. This figure shows a marked overlap, at each time point, of the biomarker level between patients who developed stage 1 CS-AKI and those who did not. The overlap was less important for changes in plasma creatinine (from end of CPB to H6). Box and Whisker plots represent the 1st and 3rd quartiles (the 25th and 75th percentiles) and the median (the 50th percentile). Outside values are represented by squares. Far out values are represented by circles.

**Supplemental Figure 2: Early detection of CS-AKI in the same population (n=59).**


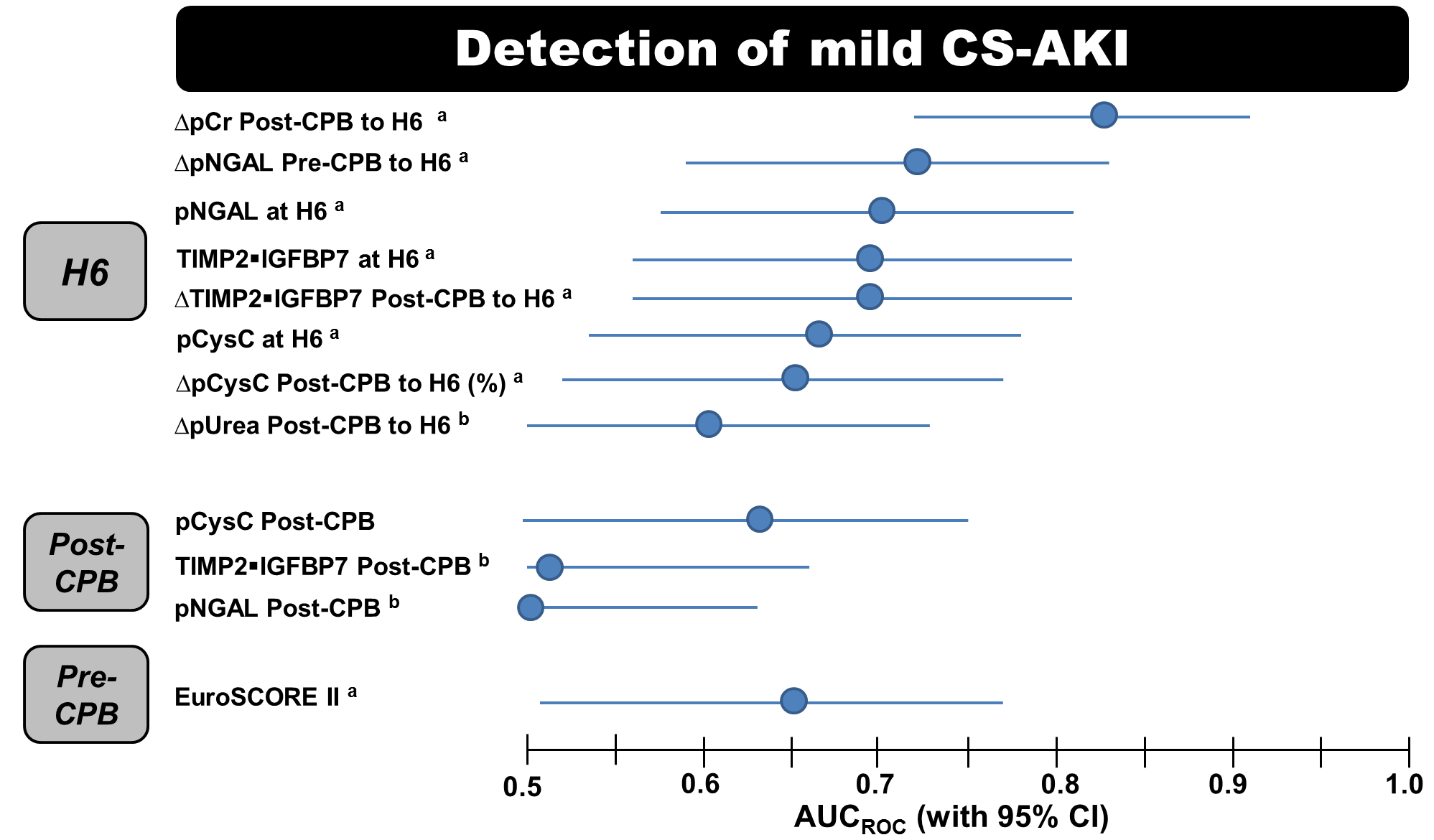


Legend: The accuracy for the prediction or the detection of stage ≥1 cardiac surgery-associated acute kidney injury (CS-AKI) was assessed via the area under receiver operating characteristic curve (AUC_ROC_ [95% confidence interval]) in the 59 patients who had all biomarkers measured.

Each biomarker was tested for 1) an isolated sample taken before (Pre-CPB), immediately after (Post-CPB) cardiopulmonary by-pass and 6 hours (H6) after the end of the surgery and 2) for change in concentration (absolute or relative [%]) between 2 time points. Only the highest AUC_ROC_ was retained for each biomarker or its change. All AUC_ROC_ values are provided in Supplemental Tables 1a to 1e.

∆: change in biomarker concentration; TIMP2▪IGFBP7: tissue inhibitor of metalloproteinase 2 ̶ insulin-like growth factor-binding protein 7; pNGAL: plasma neutrophil gelatinase-associated lipocalin; pCysC: plasma cystatin C; pCr: plasma creatinine.

^a^: p<0.05 for comparison of the AUC_ROC_ with 0.50.

^b^: p<0.05 for comparison of the AUC_ROC_ with “∆Cr Post-CPB to H6”.

All other comparisons between the listed AUC_ROC_s yielded p values >0.08.

**Supplemental Figure 3: Refining pCr-based early detection of CS-AKI with the use of a second biomarker.**

***Supplemental Figure 3a:***


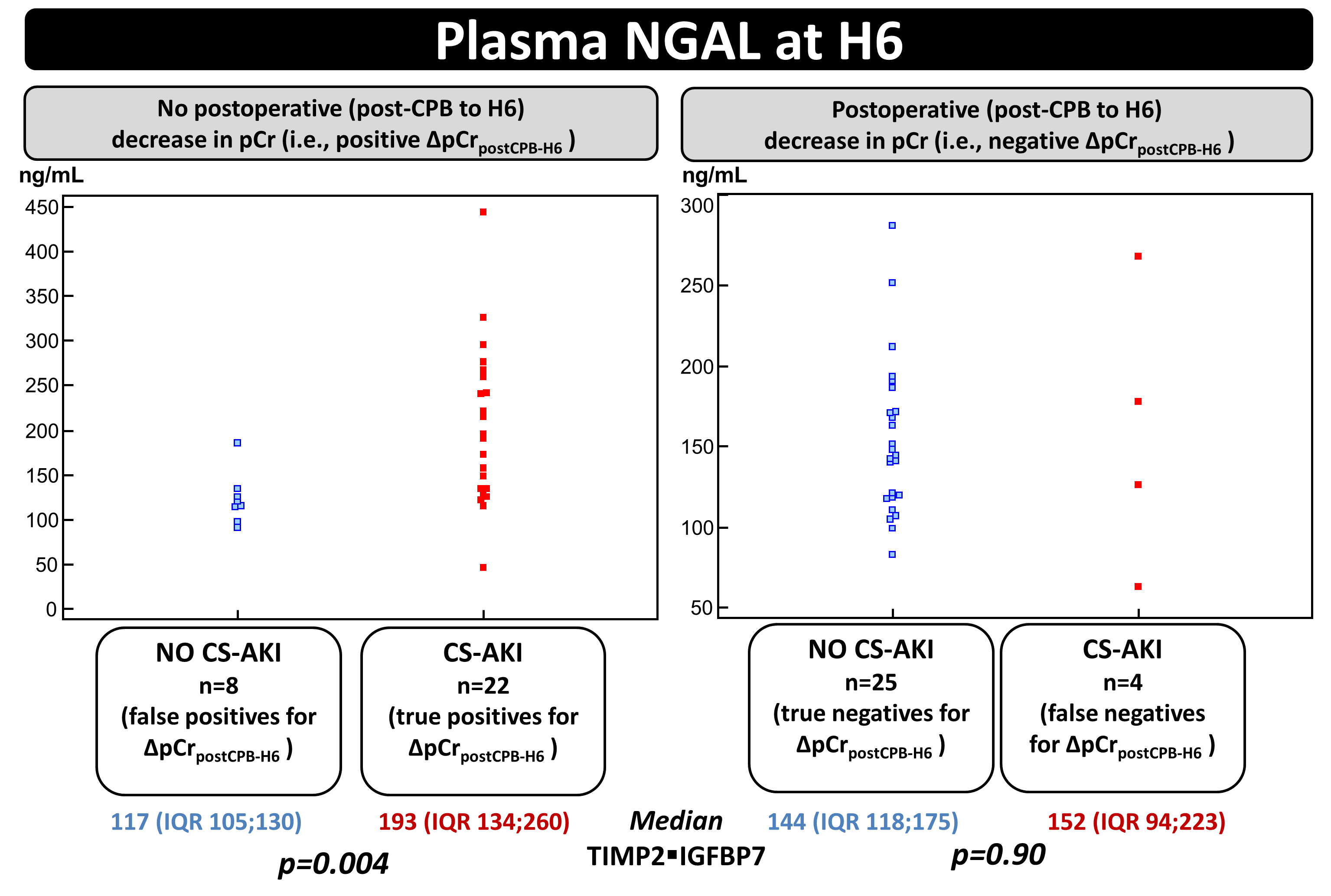


***Supplemental Figure 3b:***


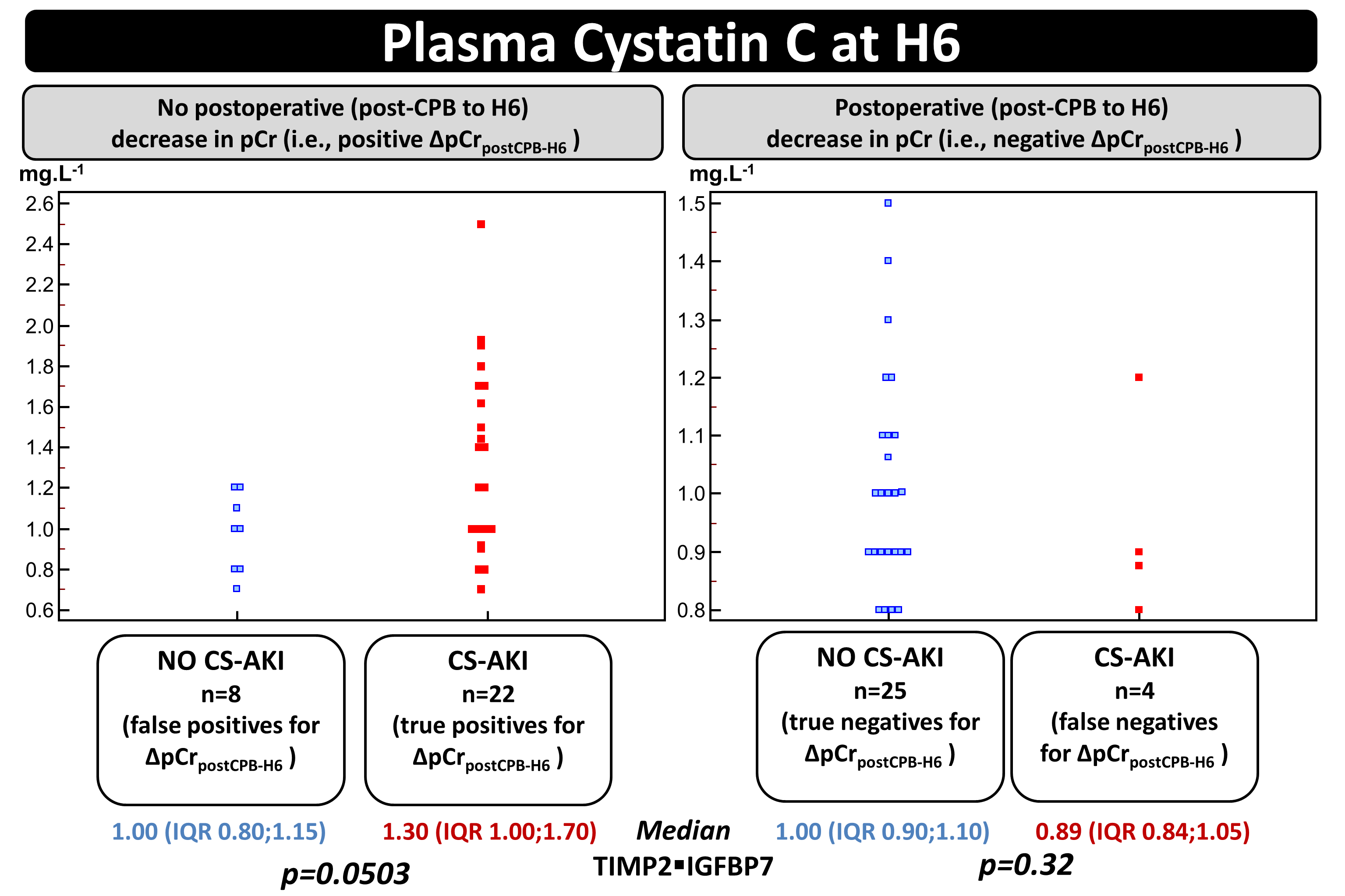


***Supplemental Figure 3c*:**


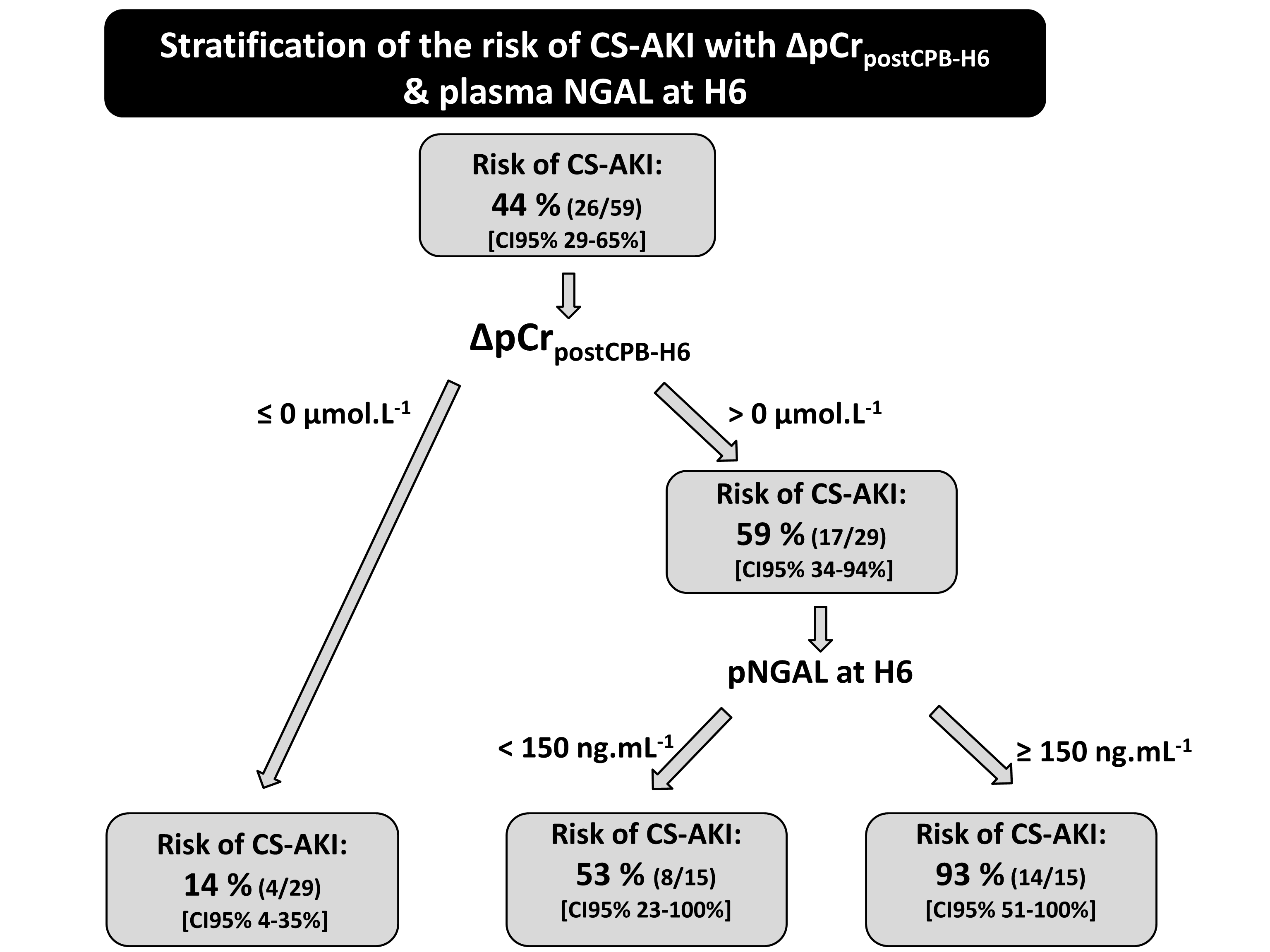


Legend: CS-AKI: Cardiac surgery-associated acute kidney injury; CPB: cardiopulmonary bypass; ∆pCr_postCPB-H6_: change in plasma creatinine from CPB to H6; NGAL: neutrophil gelatinase-associated lipocalin. CS-AKI was classified according to Kidney Disease Improving Global Outcome (KDIGO) guidelines.

These 3 figures show a means to combine pCr and novel biomarkers. For instance, if pNGAL reached a high level at H6 (>150 ng.mL^-1^) in patients with positive ∆pCr_postCPB-H6_, the occurrence of CS-AKI was likely (14 out of 15 patients [93%; 95%CI 51-100%]). This was also true, to a lesser extent, with pCysC. In other words, a marked increase in pNGAL (or pCysC) may be useful to rule out a false positivity of ∆pCr_postCPB-H6_. The ability of pNGAL or pCysC to rule out a false negativity appeared to be poor.

Overall, wide confidence intervals prevent drawing firm conclusions.

**Supplemental Figure 4: Distinction between persistent and transient cardiac surgery-associated AKI.**


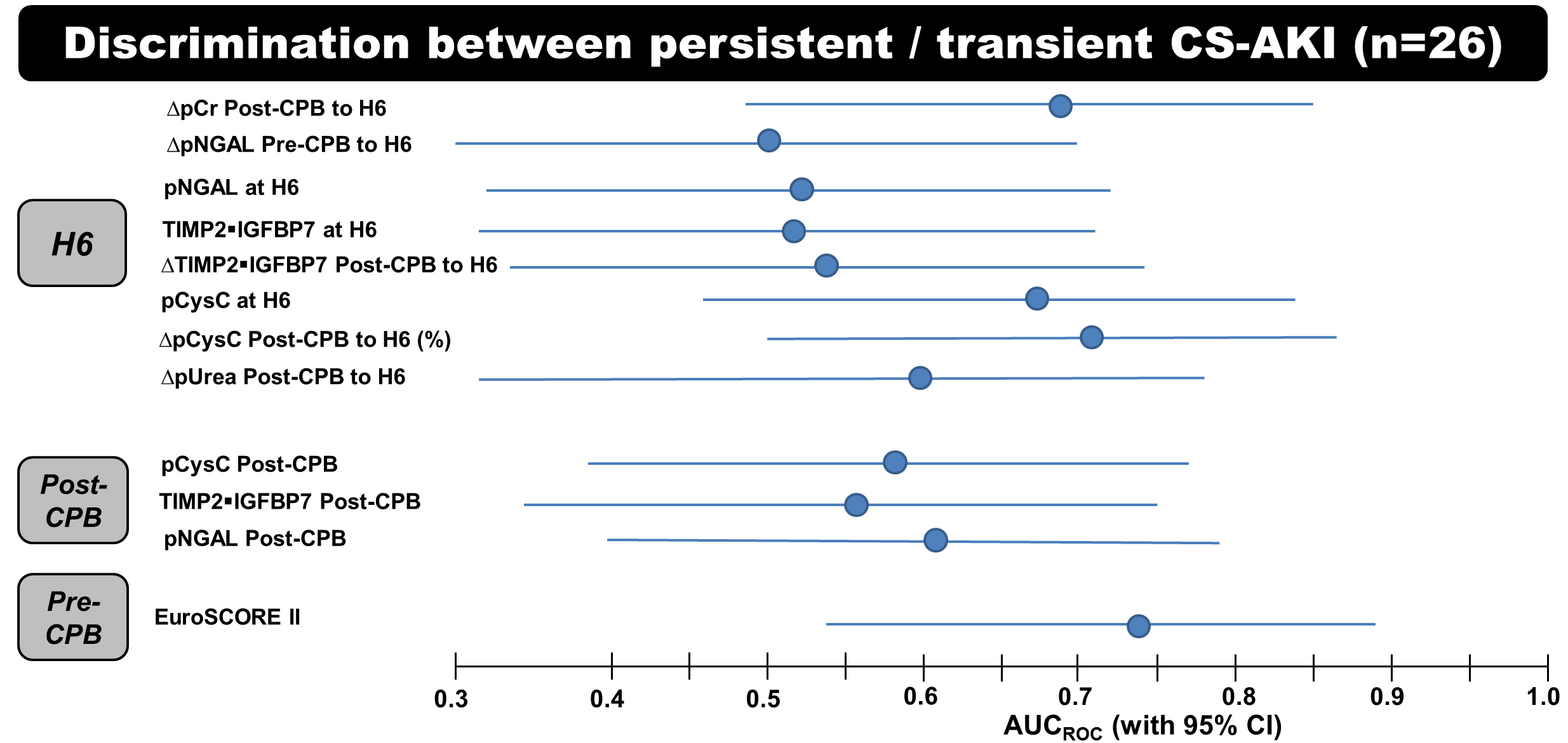


Legend: Among the 26 patients who developed cardiac surgery-associated acute kidney injury (CS-AKI), the accuracy for the early recognition of persistent CS-AKI was assessed via the area under receiver operating characteristic curve (AUC_ROC_ [95% confidence interval]).

Each biomarker was tested for 1) an isolated sample taken before (Pre-CPB), immediately after (Post-CPB) cardiopulmonary by-pass and 6 hours (H6) after the end of the surgery and 2) for change in concentration (absolute or relative [%]) between 2 time points. ∆: change in biomarker concentration; TIMP2▪IGFBP7: tissue inhibitor of metalloproteinase 2 ̶ insulin-like growth factor-binding protein 7; pNGAL: plasma neutrophil gelatinase-associated lipocalin; pCysC: plasma cystatin C; pCr: plasma creatinine.

**Supplemental Table 1: STARD checklist.**

|  | **Section & Topic** | **No** | **Item** | **Reported on page #** |
| --- | --- | --- | --- | --- |
|  |  |  |  |  |
|  | **TITLE OR ABSTRACT** |  |  |  |
|  |  | **1** | Identification as a study of diagnostic accuracy using at least one measure of accuracy  (such as sensitivity, specificity, predictive values, or AUC) | Abstract |
|  | **ABSTRACT** |  |  |  |
|  |  | **2** | Structured summary of study design, methods, results, and conclusions  (for specific guidance, see STARD for Abstracts) | Abstract |
|  | **INTRODUCTION** |  |  |  |
|  |  | **3** | Scientific and clinical background, including the intended use and clinical role of the index test | Introduction section |
|  |  | **4** | Study objectives and hypotheses | End of the introduction section |
|  | **METHODS** |  |  |  |
|  | *Study design* | **5** | Whether data collection was planned before the index test and reference standard  were performed (prospective study) or after (retrospective study) | Methods>Patients |
|  | *Participants* | **6** | Eligibility criteria | Methods>Patients |
|  |  | **7** | On what basis potentially eligible participants were identified  (such as symptoms, results from previous tests, inclusion in registry) | Methods>Patients |
|  |  | **8** | Where and when potentially eligible participants were identified (setting, location and dates) | Methods>Patients |
|  |  | **9** | Whether participants formed a consecutive, random or convenience series | Methods>Patients |
|  | *Test methods* | **10a** | Index test, in sufficient detail to allow replication | Methods>Patients |
|  |  | **10b** | Reference standard, in sufficient detail to allow replication | Methods>Measurements |
|  |  | **11** | Rationale for choosing the reference standard (if alternatives exist) | Methods>Measurements |
|  |  | **12a** | Definition of and rationale for test positivity cut-offs or result categories  of the index test, distinguishing pre-specified from exploratory | Methods>Statistical analysis |
|  |  | **12b** | Definition of and rationale for test positivity cut-offs or result categories  of the reference standard, distinguishing pre-specified from exploratory | Methods>Measurements |
|  |  | **13a** | Whether clinical information and reference standard results were available  to the performers/readers of the index test | Methods>Measurements |
|  |  | **13b** | Whether clinical information and index test results were available  to the assessors of the reference standard | Methods>Measurements |
|  | *Analysis* | **14** | Methods for estimating or comparing measures of diagnostic accuracy | Methods>Statistical analysis |
|  |  | **15** | How indeterminate index test or reference standard results were handled | NA |
|  |  | **16** | How missing data on the index test and reference standard were handled | Methods>Statistical analysis |
|  |  | **17** | Any analyses of variability in diagnostic accuracy, distinguishing pre-specified from exploratory | NA |
|  |  | **18** | Intended sample size and how it was determined | Methods>Statistical analysis>Study size |
|  | **RESULTS** |  |  |  |
|  | *Participants* | **19** | Flow of participants, using a diagram | Figure 1 |
|  |  | **20** | Baseline demographic and clinical characteristics of participants | Table 1 |
|  |  | **21a** | Distribution of severity of disease in those with the target condition | Figure 1 |
|  |  | **21b** | Distribution of alternative diagnoses in those without the target condition | NA |
|  |  | **22** | Time interval and any clinical interventions between index test and reference standard | NA |
|  | *Test results* | **23** | Cross tabulation of the index test results (or their distribution)  by the results of the reference standard | Figure 1 |
|  |  | **24** | Estimates of diagnostic accuracy and their precision (such as 95% confidence intervals) | Results section |
|  |  | **25** | Any adverse events from performing the index test or the reference standard | NA |
|  | **DISCUSSION** |  |  |  |
|  |  | **26** | Study limitations, including sources of potential bias, statistical uncertainty, and generalisability | Discussion section>Study limitations |
|  |  | **27** | Implications for practice, including the intended use and clinical role of the index test | Discussion section |
|  | **OTHER INFORMATION** |  |  |  |
|  |  | **28** | Registration number and name of registry | Not registered |
|  |  | **29** | Where the full study protocol can be accessed | NA |
|  |  | **30** | Sources of funding and other support; role of funders | Title page |
|  |  |  |  |  |

**Supplemental Table 2: Comparison of baseline characteristics of included and excluded patients.**

|  | ***Included patients***  ***n=65*** | ***Excluded***  ***patients***  ***n=33*** | ***p*** |
| --- | --- | --- | --- |
| Age (years) | 79 [77-81] | 79 [78-82] | 0.11 |
| Female gender (n [%]) | 33 (52 %) | 16 (50 %) | 0.89 |
| Body mass index (kg/m^2^) | 26 [24-29] | 27 [24-30] | 0.48 |
| Weight (kg) | 70 [63-81] | 72 [64-80] | 0.83 |
| EuroSCORE II (%) | 2.7 [1.6-5.1] | 2.6 [2.0-3.0] | 0.84 |
| SAPS II | 26 [22-29] | 26 [24-29] | 0.78 |
| Preoperative serum creatinine (µmol/L) | 80 [68-92] | 75 [59-89] | 0.41 |
| Preoperative eGFR (mL/minute/1.73 m^2^) | 72 [64-83] | 72 [59-85] | 0.77 |

**Legend**: SAPS II: simplified acute physiology score 2; eGFR: estimated glomerular filtration rate (modification of diet in renal disease equation.

Results are expressed as n (%) or median [interquartile range].

**Supplemental Table 3a: Performance of pCr for the detection of CS-AKI**

|  |  | **n** | **AUC_ROC_**  (95%CI) | **Standard error** |
| --- | --- | --- | --- | --- |
| **pCr** | **Pre-CPB** | 26/59 | 0.58  (0.44-0.71) | 0.0768 |
|  | **Post-CPB** | 26/59 | 0.56  (0.43-0.69) | 0.0766 |
|  | **H6** | 26/59 | 0.66  (0.53-0.78) | 0.0731 |
| **∆pCr** | **Pre-CPB to Post-CPB (%)** | 26/59 | 0.51  (0.37-0.64) | 0.0789 |
|  | **Pre-CPB to Post-CPB** | 26/59 | 0.52  (0.39-0.65) | 0.0789 |
|  | **Pre-CPB to H6 (%)** | 26/59 | 0.68  (0.54-0.79) | 0.0727 |
|  | **Pre-CPB to H6** | 26/59 | 0.69  (0.55-0.80) | 0.0727 |
|  | **Post-CPB to H6 (%)** | 26/59 | 0.80  (0.68-0.90) | 0.0613 |
|  | **Post-CPB to H6** | 26/59 | 0.81  (0.69-0.90) | 0.0570 |
|  | **Post-CPB to H6 & corrected with albuminemia** | 26/59 | 0.67  (0.53-0.79) | 0.0708 |

Legend: pCr: plasma creatinine; ∆: change in biomarker concentration between 2 time points. Time points were immediately before (pre-CPB) and after the cardiopulmonary bypass (post-CPB), 6 hours after the end of CPB (H6). CS-AKI: cardiac surgery-associated acute kidney injury. AUC_ROC_: area under the receiver operating characteristic curve. 95%CI: 95% confidence interval.

**Supplemental Table 3b: Performance of TIMP2▪IGFBP7 for the detection of CS-AKI**

**Legend: TIMP2IGFBP7: tissue inhibitor of metalloproteinase 2 ̶ insulin-like growth factor-binding protein 7; ∆: change in biomarker concentration between 2 time points. Time points were immediately before (pre-CPB) and after the cardiopulmonary bypass (post-CPB), 6 hours after the end of CPB (H6), the day after the surgery (Day1). CS-AKI: cardiac surgery-associated acute kidney injury. AUC_ROC_: area under the receiver operating characteristic curve. 95%CI: 95% confidence interval.**

|  |  | **n** | **AUC_ROC_**  (95%CI) | **Standard error** |
| --- | --- | --- | --- | --- |
| **TIMP2▪IGFBP7** | **Pre-CPB** | 26/59 | 0.58  (0.45-0.71) | 0.0760 |
|  | **Post-CPB** | 26/59 | 0.52  (0.39-0.66) | 0.0760 |
|  | **H6** | 26/59 | 0.69  (0.56-0.81) | 0.0704 |
|  | **H6 indexed to urine creatine** | 26/59 | 0.69  (0.55-0.80) | 0.0723 |
|  | **Day1** | 26/59 | 0.59  (0.46-0.71) | 0.0743 |
| **∆TIMP2▪IGFBP7** | **Pre-CPB to Post-CPB (%)** | 26/59 | 0.51  (0.37-0.64) | 0.0760 |
|  | **Pre-CPB to Post-CPB** | 26/59 | 0.50  (0.37-0.64) | 0.0764 |
|  | **Pre-CPB to H6 (%)** | 26/59 | 0.65  (0.52-0.77) | 0.0733 |
|  | **Pre-CPB to H6** | 26/59 | 0.68  (0.54-0.79) | 0.0728 |
|  | **Post-CPB to H6 (%)** | 26/59 | 0.66  (0.53-0.78) | 0.0748 |
|  | **Post-CPB to H6** | 26/59 | 0.69  (0.56-0.81) | 0.0732 |
|  | **Pre-CPB to H6 indexed to urine creatinine** | 26/59 | 0.66  (0.53-0.78) | 0.0737 |
|  | **Post-CPB to H6 indexed to urine creatinine** | 26/59 | 0.66  (0.52-0.77) | 0.0753 |

**Supplemental Table 3c: Performance of pCysC for the detection of CS-AKI**

Legend: pCysC: plasma cystatin C; ∆: change in biomarker concentration between 2 time points. Time points were immediately before (pre-CPB) and after the cardiopulmonary bypass (post-CPB), 6 hours after the end of CPB (H6), the day after the surgery (Day1). CS-AKI: cardiac surgery-associated acute kidney injury. AUC_ROC_: area under the receiver operating characteristic curve. 95%CI: 95% confidence interval.

|  |  | **n** | **AUC_ROC_**  (95%CI) | **Standard error** |
| --- | --- | --- | --- | --- |
| **pCysC** | **Pre-CPB** | 26/59 | 0.63  (0.49-0.75) | 0.0749 |
|  | **Post-CPB** | 26/59 | 0.63  (0.49-0.75) | 0.0760 |
|  | **H6** | 26/59 | 0.66  (0.53-0.78) | 0.0760 |
|  | **H6 & corrected with ∆albuminemia** | 26/59 | 0.65  (0.52-0.77) | 0.0748 |
|  | **Day1** | 26/59 | 0.74  (0.61-0.84) | 0.0687 |
| **∆pCysC** | **Pre-CPB to Post-CPB (%)** | 26/59 | 0.56  (0.42-0.69) | 0.0764 |
|  | **Pre-CPB to Post-CPB** | 26/59 | 0.54  (0.41-0.67) | 0.0763 |
|  | **Pre-CPB to H6 (%)** | 26/59 | 0.63  (0.49-0.75) | 0.0745 |
|  | **Pre-CPB to H6** | 26/59 | 0.62  (0.48-0.74) | 0.0750 |
|  | **Post-CPB to H6 (%)** | 26/59 | 0.65  (0.52-0.77) | 0.0732 |
|  | **Post-CPB to H6** | 26/59 | 0.64  (0.51-0.76) | 0.0738 |

**Supplemental Table 3d: Performance of pNGAL for the detection of CS-AKI**

Legend: pNGAL: plasma neutrophil gelatinase-associated lipocalin; ∆: change in biomarker concentration between 2 time points. Time points were immediately before (pre-CPB) and after the cardiopulmonary bypass (post-CPB), 6 hours after the end of CPB (H6), the day after the surgery (Day1). CS-AKI: cardiac surgery-associated acute kidney injury. AUC_ROC_: area under the receiver operating characteristic curve. 95%CI: 95% confidence interval.

|  |  | **n** | **AUC_ROC_**  (95%CI) | **Standard error** |
| --- | --- | --- | --- | --- |
| **pNGAL** | **Pre-CPB** | 26/59 | 0.58  (0.45-0.71) | 0.0757 |
|  | **Post-CPB** | 26/59 | 0.50  (0.37-0.64) | 0.0833 |
|  | **H6** | 26/59 | 0.70  (0.57-0.81) | 0.0711 |
|  | **H6 & corrected with ∆albuminemia** | 26/59 | 0.69  (0.56-080) | 0.0718 |
|  | **Day1** | 29/59 | 0.70  (0.57-0.81) | 0.0735 |
| **∆pNGAL** | **Pre-CPB to Post-CPB (%)** | 26/59 | 0.57  (0.43-0.70) | 0.0761 |
|  | **Pre-CPB to Post-CPB** | 26/59 | 0.54  (0.41-0.67) | 0.0763 |
|  | **Pre-CPB to H6 (%)** | 26/59 | 0.63  (0.49-0.75) | 0.0730 |
|  | **Pre-CPB to H6** | 26/59 | 0.72  (0.59-0.83) | 0.0689 |
|  | **Post-CPB to H6 (%)** | 26/59 | 0.71  (0.58-0.82) | 0.0691 |
|  | **Post-CPB to H6** | 26/59 | 0.65  (0.52-0.77) | 0.0780 |

**Supplemental Table 3e: Performance of pUrea for the detection of CS-AKI**

Legend: pUrea: plasma urea; ∆: change in biomarker concentration between 2 time points. Time points were immediately before (pre-CPB) and after the cardiopulmonary bypass (post-CPB), 6 hours after the end of CPB (H6), the day after the surgery (Day1). CS-AKI: cardiac surgery-associated acute kidney injury. AUC_ROC_: area under the receiver operating characteristic curve. 95%CI: 95% confidence interval.

|  |  | **n** | **AUC_ROC_**  (95%CI) | **Standard error** |
| --- | --- | --- | --- | --- |
| **pUrea** | **Pre-CPB** | 26/59 | 0.51  (0.38-0.64) | 0.0773 |
|  | **Post-CPB** | 26/59 | 0.51  (0.38-0.64) | 0.0773 |
|  | **H6** | 26/59 | 0.52  (0.39-0.65) | 0.0777 |
|  | **H6 & corrected with ∆albuminemia** | 26/59 | 0.52  (0.39-0.66) | 0.0774 |
|  | **Day1** | 26/59 | 0.65  (0.51-0.77) | 0.0758 |
| **∆pUrea** | **Pre-CPB to Post-CPB (%)** | 26/59 | 0.51  (0.37-0.64) | 0.0760 |
|  | **Pre-CPB to Post-CPB** | 26/59 | 0.50  (0.37-0.64) | 0.0764 |
|  | **Pre-CPB to H6 (%)** | 26/59 | 0.65  (0.52-0.77) | 0.0733 |
|  | **Pre-CPB to H6** | 26/59 | 0.68  (0.54-0.79) | 0.0728 |
|  | **Post-CPB to H6 (%)** | 26/59 | 0.66  (0.53-0.78) | 0.0748 |
|  | **Post-CPB to H6** | 26/59 | 0.69  (0.56-0.81) | 0.0732 |
|  | **Post-CPB to H6 & corrected with ∆albuminemia** | 26/59 | 0.56  (0.42-0.69) | 0.0773 |

**Supplemental Table 4: Performance for the prediction or detection of CS-AKI according to the definition (omitting the urine output criterion or not) [n=59].**

|  | **DETECTION of CS-AKI :**  **definition including pCr AND urine output** | | **DETECTION of CS-AKI :**  **definition only including pCr** | |
| --- | --- | --- | --- | --- |
|  | **AUC** | **SE** | **AUC** | **SE** |
| **∆pCr**  **POST CPB to H6** | 0.79  (0.65-0.88) | 0.0657 | 0.90  (0.80-0.97) | 0.0402 |
| **TIMP2▪IGFBP7 at H6** | 0.67  (0.53-0.79) | 0.0752 | 0.67  (0.53-0.78) | 0.103 |
| **∆TIMP2▪IGFBP7**  **POST CPB to H6** | 0.68  (0.54-0.80) | 0.0787 | 0.61  (0.48-0.74) | 0.114 |
| **pCysC at H6** | 0.63  (0.49-0.75) | 0.0834 | 0.78  (0.65-0.88) | 0.0748 |
| **∆pNGAL**  **PRE CPB to H6** | 0.68  (0.54-0.80) | 0.0760 | 0.74  (0.61-0.85) | 0.0793 |
| **∆pUREA**  **PRE CPB to H6** | 0.58  (0.44-0.71) | 0.0811 | 0.67  (0.54-0.79) | 0.0903 |

Legend: cardiac surgery-associated acute kidney injury (CS-AKI); ∆: change in biomarker concentration; TIMP2▪IGFBP7: tissue inhibitor of metalloproteinase 2 ̶ insulin-like growth factor-binding protein 7; pNGAL: plasma neutrophil gelatinase-associated lipocalin; pCysC: plasma cystatin C; pCr: plasma creatinine.

With the full definition of CS-AKI, 26 patients (44%) developed CS-AKI. With the definition of CS-AKI only including pCr, 10 patients (17%) developed CS-AKI.
